# Assessing implementation modalities of mhealth intervention on pregnant women in Dschang health district, West region of Cameroon

**DOI:** 10.11604/pamj.2019.33.305.17603

**Published:** 2019-08-19

**Authors:** Nkemngu Blake Afutendem, Aubin Nino Baleba, Azefack Léon Tapondjou, Claude Ngwayu Nkfusai, Vecheusi Zennobia Viyoff, Frankline Sanyuy Nsai, Joyce Shirinde, Samuel Nambile Cumber

**Affiliations:** 1 Department of Public Health, Faculty of Medicine and Pharmaceutical sciences, University of Dschang, Dschang, Cameroon; 2 HIV Free Project, Cameroon Baptist Convention Health Services, Yaounde, Cameroon; 3 Department of Microbiology and Parasitology, Faculty of Science, University of Buea, Buea, Cameroon; 4 School of Health Systems and Public Health, Faculty of Health Sciences, University of Pretoria Private Bag X323, Gezina, Pretoria, 0001, Pretoria, South Africa; 5 Faculty of Health Sciences, University of the Free State, Bloemfontein, South Africa; 6 Section for Epidemiology and Social Medicine, Department of Public Health, Institute of Medicine (EPSO), The Sahlgrenska Academy at University of Gothenburg, Gothenburg, Sweden

**Keywords:** Pregnant women, implementation Modalities, mHealth, Dschang health district, West Region, Cameroon

## Abstract

**Introduction:**

Every 90 seconds, a woman dies of complications related to pregnancy and childbirth, resulting in more than 340,000 maternal deaths a year. Antenatal care (ANC) and postnatal care (PNC) are significant determinants of maternal health and, particularly, safe motherhood. Antenatal care is an important predictor of safe delivery and provides health information and services that can improve the health of women and infants. mHealth broadly encompasses the use of mobile telecommunication and multimedia technologies as they are integrated within increasingly mobile and wireless health care delivery systems. This study aimed at assessing the acceptable implementation modalities of mHealth intervention on pregnant Women in Dschang health district, West Region of Cameroon.ng ba.

**Methods:**

This was a cross sectional descriptive study in the Dschang health district, West region of Cameroon. Key informants were all pregnant women from 18 years and above and a total of 372 pregnant women were included. This study was carried out from March to July 2017.

**Results:**

Majority of the women, that is, 252(67.74%) were married, 117(31.45%) declaredtheir status as being single, while 3(0.81%) were devorced. Out of the 335 women that declared wanting an mHealth intervention, 41.79% of this number preferred SMS texts in the afternoon, 111(33.13%) in the evening, 46(13.73%) anytime and 38(11.34%) in the morning hours. A total of 83.33% women confirmed using telephone services.

**Conclusion:**

This study reveals that cell phones would be the acceptable medium of providing pregnancy and postpartum support to women in the Dschang health district. This is justified by the fact that a vast majority of women interviewed had access to a cell phone and referred to it as their desired and accepted means of communication.

## Introduction

mHealth broadly encompasses the use of mobile telecommunication and multimedia technologies as they are integrated within increasingly mobile and wireless health care delivery systems [1]. The field broadly incorporates the use of mobile telecommunication and multimedia technologies in health care delivery [2]. The term mHealth was coined by Robert Istepanian as use of “emerging mobile communications and network technologies for healthcare.” A definition used at the 2010 mHealth Summit of the Foundation for the National Institutes of Health (FNIH) was *"the delivery of healthcare services via mobile communication devices,"* [3]. mHealth broadly encompasses the use of mobile telecommunication and multimedia technologies as they are integrated within increasingly mobile and wireless health care delivery systems [4]. The field broadly encompasses the use of mobile telecommunication and multimedia technologies in health care delivery [5]. The term mHealth was coined by Robert Istepanian as use of “emerging mobile communications and network technologies for healthcare”. A definition used at the 2010 mHealth Summit of the Foundation for the National Institutes of Health (FNIH) was *"the delivery of healthcare services via mobile communication devices"* [1]. While there are some projects that are considered solely within the field of mHealth, the linkage between mHealth and eHealth is unquestionable [1]. For example, a mHealth project that uses mobile phones to access data on HIV/AIDS rates would require an eHealth system in order to manage, store, and assess the data. Thus, eHealth projects operate many times as the backbone of mHealth projects [6]. Antenatal care (ANC) and postnatal care (PNC) are significant determinants of maternal health and, particularly, safe motherhood. Antenatal care is an important predictor of safe delivery and provides health information and services that can improve the health of women and infants [2]. In addition, ANC has a positive impact on the utilization of postnatal healthcare services, while PNC and intrapartum (the period from the onset of labour to the end of the third stage of labour) care significantly reduces maternal mortality given that most maternal deaths occur in the first week after delivery [2].

Every 90 seconds, a woman dies of complications related to pregnancy and childbirth, resulting in more than 340,000 maternal deaths a year. Millions of women suffer from pregnancy-related illnesses or experience other severe consequences such as infertility, fistula and incontinence [1]. Delay is considered the key factor responsible for women not accessing health services. There are three phases of delay: (i) recognizing the need for health care and in the decision making process; (ii) arrival at a health facility; and (iii) receiving appropriate and adequate care at the health facility [7]. Underlying determinants that cause the delays are the position of women in society, long geographical distances, weak health systems, poverty and lack of education [1,6]. There is an extensive agreement that access to communication is an essential component of improving the use and quality of maternal health services. Several studies have proven the effectiveness of the mobile phone technology in the amelioration of mother and child healthcare through its use in the amelioration of antenatal care uptake in health facilities. Antenatal care (ANC) and postnatal care (PNC) are significant determinants of maternal health and, particularly, safe motherhood. Antenatal care is an important predictor of safe delivery and provides health information and services that can improve the health of women and infants [2]. In addition, ANC has a positive impact on the utilization of postnatal healthcare services, while PNC and intrapartum (the period from the onset of labour to the end of the third stage of labour) care significantly reduces maternal mortality given that most maternal deaths occur in the first week after delivery [2]. The mobile phone has a high potential as it is small, portable, widely used, relatively cheap and its extended network coverage increasingly enables communication with rural and isolated areas [8]. This technology enhances components like: accessing emergency obstetric care, improving capacity of lesser trained health staff, and empowering women to contact health services and access information [8]. This study aims at assessing the acceptable implementation modalities of mHealth intervention on pregnant Women in the Dschang Health district, West Region of Cameroon.

## Methods

**Study design:** this was a cross sectional descriptive study.

**Study area:** the Dschang health district, West region of Cameroon.

### Selection criteria

**Inclusion criterion:** pregnant women, with the minimum 18 years old who gave their consent to participate in the study.

**Exclusion criteria:** pregnant women with less than 18 years and pregnant women who did not give consent to participate in the study; pregnant women who refuse to continue participating in the study after consent (inconvenient questions).

**Sample size:** sample size was determined using the Fisher’s formula;

$$N=\frac{Z^{2}*P(1-P)}{e^{2}}$$

N = minimum sample size, Z = Z–value corresponding to 95% confidence interval (1.96), P = The acceptable implementation modalities of mHealth intervention on Pregnant Women in Dschang Health district, West Region of Cameroon is not known; hence 50% will be used, e = error margin (0.05). Therefore, N= 1.96²*0.05 (1-0.05)/0.05² N=372. From the calculation, the minimum sample size is 372 pregnant women to be included.

**Sampling methods:** the recruitment centre was the Dschang health district Hospital. This choice was made out of convenience because they account for the greatest number of prenatal consultations in the Dschang health district. Investigators interviewed all eligible women who had consented to participate in the study until the minimum sample size number was reached.

**Data collection and analyses:** the data were collected with the use of questionnaires, pretested by some 15 female students in the department of Public Health of the university of Dschang, and made available in English (original language which the questionnaire was designed) and translated into French by a final year master’s student in Bilingual Letters of the University of Dschang. This questionnaire was adapted from a previous study by Cormick and colleagues in Argentina and the main question was on modalities of mHealth intervention on pregnant Women [9]. They wereorally administered by the investigator after prenatal consultations had been done with the selected women who had consented to participate in the study. The period of data collection was one month, one week (from the 30^th^ of May to the 7^th^ of July 2017), and was done in the Dschang district hospital. There are pre organised sessions of prenatal consultations on every Mondays, so, special emphasis and mobilisation was done every Monday to administer questionnaires to pregnant women. Sampling was performed by convenience. Investigators recruited all eligible women who consented to participate until the minimum sample size number was reached. The questionnaires were then coded and entered in EPI Info 7.1.3.3 and thus analysed. Descriptive and inferential statistical analyses were used.

**Data quality assessment:** questionnaires were checked by the principal investigator for completeness on a daily basis by immediate supervisors. After checking for consistency and completeness, the supervisor submitted the filled questionnaire to the principal investigator. Incorrectly filled or missed ones were sent back to respective data collectors for correction. The principal investigator again rechecked the completed questionnaires to maintain the quality of data.

**Ethical considerations:** given the fact that this research involved humans (participants, investigator, data collectors), ethical consideration was mandatory. In this light, ethical clearance was obtained from the National Ethical Committee in Yaoundé (Ref: 446/17).

**Potential risk minimization:** the participants of this research (community individuals of all ages) have some potential risk link to this study which includes: violation of autonomy, rupture of confidentiality on the private data with regards to participants, exploitation of participants and inequality in risk/benefit ratio and also rupture of social equilibrium in the community. Such potential risks were minimized by all the information mentioned in the information notice. These risks were minimized in the following ways: administering Informed Consent to participants before administering questionnaire; Respecting the autonomy of participants; The data collection tool (questionnaire) were anonymous, and access to data was restricted only to those concerned, physical and electronic barriers; Electronic data were stored in an apple cloud account accessible only to the principal investigator and the physical questionnaires burned after verification of data; Permission was obtained both from the District administration and the hospital administration.

## Results

**Socio demographic characteristics:** the average age of women used in this study was 26.9(min 18, max 40). A total of 175(47%) of the women had the secondary school as their highest level of education, while 3(0.81%) women declared that they didn’t go to school. Also, while 124 (33.33%) women were students (level undifferentiated), and were the highest represented, they were followed by business women with 108(29.03%). The least represented were farmers with 15(4.03%). Majority of the women, that is 252 (67.74%) were married, 117(31.45%) of the women asserted being single, while 3(0.81%) were divorced. In addition, while 336(90.32%) of the women were Christians, 28(7.53%) were Muslim and 8(2.15%) admitted being pagans.

**Figure 1:**
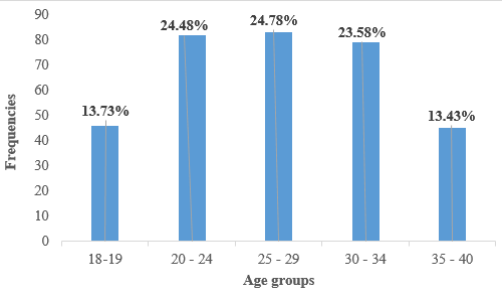
Distribution of participants willing to accept mhealth intervention per age group

**Interest of pregnant women in the use of SMS texts and voice calls in the improvement of perinatal and postnatal care:** a total of 335(90.05%) women were willing to receive SMS messages (pre and post-partum) against 37(9.95%) who weren’t ready. Their interest in the use of SMS texts and voice calls in the improvement of perinatal and postnatal care is distributed per age group as shown in Figure 1.

**Figure 2:**
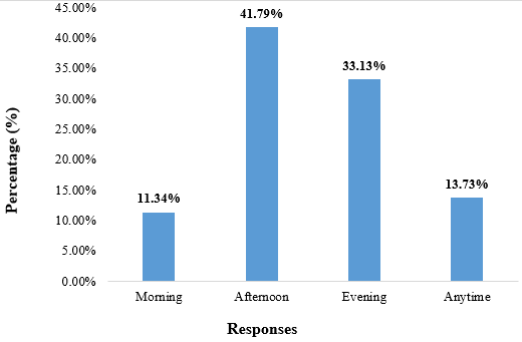
Preferred time of the day pregnant women will be receptive to SMS text messages and phone calls

**Time of the day they wish to receive SMS texts and voice calls:** for the 335 women that affirmed wanting a mHealth intervention, 41.79% wanted to receive calls in the afternoon, 33.13% in the evening, 13.73% at any time and 11.34% in the morning (Figure 2).

**Figure 3:**
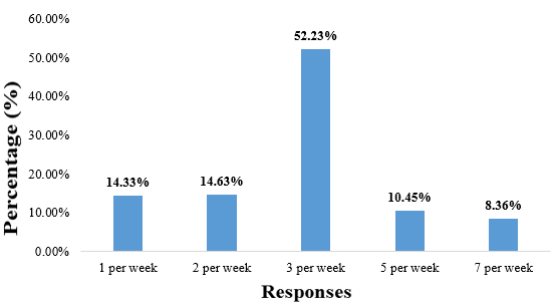
Preferred frequency of SMS texts and phone calls by pregnant women

**Women’s preferred frequency of SMS or phone calls:** a staggering 52.23% preferred SMS or phone calls at a rate of 3 per week. The distribution of these frequencies is better illustrated in Figure 3.

**Network operators:** a total of 310(83.33%) women affirmed using MTN, followed by Orange with 250(67.2%), and last by NEXTTEL with 105(28.23%) women.

## Discussion

Majority of the women (90.05%) were willing to receive SMS messages and phone calls during and after their pregnancy, which is less than the 96% obtained by Cormick *et al*. in 2012 [9], but still very close. We can also observe that the age group that holds the majority of positive responses to wanting a possible mHealth intervention is the 25-29 years old age group. Most of the women wished to receive their SMS messages in the afternoon (41.7%) and evening (33.13%) meaning that in an eventuality of a mHealth program, these are the times the women will be more receptive to receiving information. This again differs from the results obtained by [9] stating that majority (36.3%) of the women wished to receive this information anytime. To say more, 52.23% of the pregnant women interviewed wished to receive 3 messages per week, which is different from the results obtained by [9], stating that a majority (52.7%) of women wished to receive one SMS per day. All of this only goes to prove how different communities could be and that operational research should be done before, during and after any program implementation in every community. A potential drawback to implementing a text-messaging program is that it requires the recipient to have an adequate level of literacy, and marginalizing groups who could potentially benefit from the intervention. In our study population, this could affect around 0.81% of women having no or incomplete primary schooling, against the 17% obtained by [9]. That is why these researchers thought of an SMS and voice call program. In case the woman is uneducated, the voice call option using a hotline could be used to propagate the health information needed accordingly. The high level of literacy observed in this study just comes to confirm, the regional level of literacy of girls (99%), and at the national level (87%) [10].

## Conclusion

This study reveals that cell phones would be an acceptable approach to providing pregnancy and postpartum support to women in the Dschang health district, since the vast majority of women interviewed had access to a cell phone and referred to it as their desired and accepted means of communication. In this cell phone approach, free SMS messages and voice calls will be privileged over internet based interventions.

### What is known about this topic

- Postnatal care: opportunities for Africa’s new-borns;
- Giving cell phones to pregnant women and improving services may increase primary health facility utilization;
- Interest of pregnant women in the use of SMS (short message service) text messages for the improvement of perinatal and postnatal care.

### What this study adds

- Cell phones would be an acceptable approach to providing pregnancy and postpartum support to women, since the vast majority of women interviewed had access to a cell phone and referred to it as their desired and accepted means of communication.

## Competing interests

The authors declare no competing interests.
